# Career urgency and turnover intention among young adult workers: a comparison by gender and employment status

**DOI:** 10.1186/s40359-023-01434-6

**Published:** 2023-11-11

**Authors:** Hiromi Ono

**Affiliations:** 1 Department of Psychology, Meisei University, Hodokubo, Hino-shi, Tokyo, 191-8506 Japan; 2 Faculty of Human Sciences, University of Tsukuba, Otsuka, Bunkyo-ku, Tokyo, 112-0012 Japan

**Keywords:** Career urgency, Turnover intention, Male employees, Female employees, Regular employees, Non-regular employees

## Abstract

**Background:**

This study aimed to analyze, by grouping young adult workers by gender and employment status, the model that states that the tendency for impatience in situations such as “stagnation in career exploration,” “low evaluation from affiliation,” “upward comparison of careers between friends and acquaintances,” and “lack of work–life balance” leads to turnover intention through career urgency such as the “feeling of being pressurized,” having the “urge to develop one’s career,” and having “concern for one’s career.”

**Methods:**

An online survey was conducted targeting 400 young adult workers. A simultaneous multi-population analysis was performed.

**Results:**

For both male and female regular employees, the tendency for impatience when their career exploration stagnated led to their turnover intention by the “feeling of being pressurized.” However, for both male and female non-regular employees, although the tendency for impatience promotes the “feeling of being pressurized” upon stagnation in career exploration, it does not lead to turnover intention. Further, the results showed that in the case of female non-regular employees, the tendency for impatience when comparing their own career to those of friends and acquaintances, who are in a more desirable state than their own, leads to turnover intention through “concern for one’s career.”

**Conclusions:**

Future research should consider marital status and the presence or absence of children in addition to gender and employment. Future studies should consider whether non-regular employees are of the involuntary type and whether they wish to change their status as regular employees.

## Background

In Japan, the seniority system and lifetime employment were reviewed after the collapse of the bubble economy, whereas the performance-based system only emerged in the latter half of the 1990s. The workers were required to improve their employability [1], manage and be responsible for their own careers, as well as proactively develop them. Europe and the United States are leading the way in these changes. Hall [2] argued that the structural reforms in the industrial society of the 1980s changed the psychological contract between individuals and corporate organizations. He advocated for the idea of a protean career, which is formed not by an organization but by an individual whose direction is changed each time to meet their own needs. Furthermore, Arthur and Rousseau [3] proposed a new career concept called a boundaryless career, in which careers are not limited within the boundaries of a single company, as opposed to the traditional intracompany career. Moreover, new career concepts such as the self-management of one’s career [4], which involves an individual continuously collecting information to solve career problems, have been proposed one after another. In Japan, conducting a self-analysis and setting future goals became a requirement in relation to job hunting activities [5]. In addition to this requirement, after joining a company, the employees are not only expected to improve their abilities and skills and produce results, but also required to take the initiative in shaping their careers in an autonomous manner [6]. The gap between the goals and expectations that employees set before joining a company and their present situation, as well as the inability to find clear goals and aptitudes after joining a company, has been said to create a sense of career urgency and influence the turnover intention [5].

Currently, as most new graduates are hired in Japan, the employment rate of university and graduate school graduates exceeds 90% [7]. However, the employment rate of young adult workers who have graduated from university or graduate school and are no longer students enrolled in a university or graduate school exhibits that approximately 16% have worked as workers other than regular employees, and approximately 40% have applied for jobs as a regular employee but not been hired [8]. Previous research exhibits that if a new graduate cannot secure a job with good working conditions, switching to a job with better conditions will be more difficult in Japan, as the job change market is inactive [9], and their income will likely be low thereafter [10]. The university enrollment rate in Japan has increased to over 50% for both males and females [11]. However, in relation to the occupations of young adult workers who have graduated from university or graduate school, there has been a decline in clerical work among males and an increase in blue-collar jobs, which exhibits that more people are being employed in occupations that people who have graduated from university or graduate school rarely took up in the past [12]. Moreover, the division of roles within clerical work is based on the gender role division, in which men are employed in managerial roles or positions that will lead to being a part of management in the future, whereas women are employed in non-managerial roles. This can be inferred to have changed into the differentiation of workers into male and female regular and female non-regular employees [12]. One of the characteristics of women who tend to become reluctant non-regular employees is that they were most likely temporary clerical workers in their first jobs [13].

Since approximately 30% of university graduates in Japan leave their jobs within three years of joining the company [14] and the prevention of early turnover is a challenge in many companies [15], research has been conducted on the career urgency and turnover intention among regular young adult workers. What kind of emotion is urgency? In the clinical field, research has focused mainly on behavioral aspects; for instance, “sense of time urgency,” “aggression and hostility,” and “impatience” have been pointed out as characteristics of Type A behavioral patterns [16]. In addition, in studies of depression and other disorders, urgency and anxiety are often discussed without a clear distinction between the two; psychiatrist Sullivan’s definition of anxiety includes all emotional distress [17]. In other words, from the perspective of Sullivan’s concept of anxiety, urgency can be seen as encompassing anxiety. Lazarus [18], however, states that depression is composed of anxiety, anger, guilt, and shame, and lists apprehension, unease, concern, and worry as synonymous with anxiety. Ono and Yukawa [5] consider anxiety to be a vague mood, while urgency has a particularly strong element of emotion and is considered to contain the energy that spurs action.

According to Ono and Okada [19], the young adult workers’ career urgency is divided into three aspects: the “feeling of being pressurized,” which is a feeling of hopelessness; the “urge to develop one’s career,” which is a feeling of wanting to quickly build a career to reach a goal; and the “concern for one’s career,” which are the concerns workers have about their current self or future career. According to Ono and Okada [19], career urgency is defined as “emotions, including cognitive aspects, consisting of the ‘feeling of being pressurized,’ the ‘urge to develop one’s career,’ and the ‘concern for one’s career’ arising from how one perceives oneself in the present within a temporal perspective of the past and future of one’s career.” Ono and Okada [19] used the anxiety scale [20] and 12-Item General Health Questionnaire (GHQ-12) [21], both of which measure the degree of mental health, to examine the validity of the Career Urgency Scale. The former is a scale that measures two aspects of anxiety, consisting of negative anxiety, which has a negative effect on personal growth, and positive anxiety, which promotes personal growth and self-actualization. The “Feeling of being pressurized” is a subscale of career urgency and is highly correlated with negative anxiety and GHQ-12, while another subscale, the “urge to develop one’s career,” is highly correlated with positive anxiety. This shows that the “feeling of being pressurized” is a negative aspect of career urgency, whereas the “urge to develop one’s career” is a positive aspect [19]. Further, the “urge to develop one’s career” is positively correlated with the “motivation for career development,” which is a psychological aspect of career self-reliance, and the “career development behavior” and “networking behavior” are career self-reliance behaviors [22]. Career urgency in this study follows the definition of Ono and Okada [19], which includes career anxiety and career stress as related concepts. As Ono and Yukawa [5] pointed out that urgency has a strong element of emotion, I will consider that career urgency has more inherent energy to spur action compared to these similar concepts.

In terms of the relationship between career urgency and turnover intention, research has shown that even within career urgency, the “feeling of being pressurized” promotes turnover intention [15, 23]. Furthermore, while for young adult workers, the “feeling of being pressurized” and the “urge to develop one’s career” encourages turnover activities, the “concern for one’s career” suppresses it [24, 25]. In terms of factors that influence the career urgency among young adult workers, leader–member exchanges suppress the “feeling of being pressurized,” whereas team member exchanges suppress the “feeling of being pressurized” as well as the “concern for one’s career” [25]; having a multifaceted perspective alleviates the “feeling of being pressurized” [26]. Factors that promote multifaceted perspectives include “setting and working on short-term goals,” the “acquisition of skills and knowledge through self-study,” “organization of one’s thoughts and current situation,” “career consultation,” and “meaningful work experience” [26].

However, there is scant research that focuses on the psychology of non-regular young adult workers. The involuntary non-regular employees who “wanted to work as a regular employee, but no companies were willing to hire them” are the most stressed compared to regular employees or voluntary non-regular employees [27]. However, a study by Matsuyama [28] on workers who are engaged in welfare work found no effect of their employment status on their mental health. Matsuyama [28], who examined the relationship between employment status and organizational commitment, found that non-regular employees have stronger affective and internalized factors of organizational commitment than regular employees do. The former is a concept similar to Allen and Meyer’s [29] affective commitment, which indicates an emotional attachment to the organization. The latter indicates that the organization’s values match the employees’ personal values and that they want to work for the organization [28]. Another study shows that regular employees have higher affective commitment than non-regular employees do [30]. Thus, no unified view has been obtained on the comparison of the employment types with regards to the psychology of workers. In addition, the characteristics of young people are unclear because the studies handle a wide range of age groups simultaneously.

Therefore, in this study, the researchers will examine the differences in young adult workers’ career urgency and turnover intention depending on their gender and employment status. In Japan, many women leave their jobs after childbirth, whereas it is still common for men who are raising children to have long working hours [31]. It has been suggested that more diverse factors mediate career development for women than for men [32]. Moreover, research has shown that the emergence of career urgency-provoking situations differs with regard to gender [33]. This exhibits that the possibility of leaving and changing jobs as well as the situation of career development differ, not only by employment type but also by gender. This study also examines the relationship between career urgency and turnover intention based on Ono’s [15] model. The purpose of this study was to analyze, by grouping employees in accordance with gender and employment status, the model that states that the tendency for impatience in situations such as “stagnation of career exploration,” “low evaluation from affiliation,” “upward comparison of the careers of friends and acquaintances,” and “lack of work–life balance” leads to turnover intention through career urgency such as the “feeling of being pressurized,” “urge to develop one’s career,” and “concern for one’s career.” By examining whether a difference exists in the relationship between career urgency and turnover intention depending on gender and employment status, suggestions for career support for young adult workers and retention measures in companies can be obtained.

## Methods

### Survey participants and procedures

An online questionnaire survey was conducted using Cross Marketing Group Inc. Owing to the fact that people under the age of 35 are considered young people according to various statistics of the Ministry of Health, Labor and Welfare of Japan, the participants were regular and non-regular employees (contract employees, dispatched employees) under the age of 35 who graduated from a university or graduate school and are employed by private companies in Japan. Further, considering the possibility of career urgency being a symptom of depression that appears in individual careers, those who answered “Applicable for at least one week (more than half of the period)” or “Applicable almost every day” to any of the items with regard to the two-item Patient Health Questionnaire (PHQ-2) depression screening tool were excluded. A total of 400 valid samples were collected to secure approximately 100 samples each for male regular employees, female regular employees, male non-regular employees, and female non-regular employees (mean age = 30.4 years, SD = 2.84). Table 1 shows the attributes of the respondents. The survey period was from August 8, 2022, to August 29, 2022.

**Table 1:** Respondents’ attributes by gender and employment status

	Male; 182 persons (45.5%)	Female; 218 persons (54.5%)
Regular employees; 222 persons (55.5%)	111 persons	111 persons
Non-regular employees; 178 persons (44.5%)	71 persons	107 persons

### Survey items

Turnover intention: To measure the present degree of the turnover intention, participants were asked to answer the question “How likely are you to quit your job or change jobs now? Please select one of the following that best applies to you” on a five-point scale ranging from 1 (not at all) to 5 (very much so).

Career urgency: The 16-item Career Urgency Scale of Ono and Okada [19] was used in this regard. This scale consists of three subscales, namely, the “feeling of being pressurized” (six items such as “I cannot help but feel stuck”), the “urge to develop one’s career” (five items such as “I cannot help but want to move forward”), and “concern for one’s career” (five items including “I feel frustrated with my current self”). The instruction consisted of the following: “How do you feel about your career now (including not only your specific work history but also what it means to work in your life and the image of your future work–life)? Read each of the statements below and select one of the options to indicate how much it applies to you.” Participants were asked to answer on a five-point scale ranging from 1 (does not apply) to 5 (applies).

The Career Urgency-Provoking Situation Scale: The Career Urgency-Provoking Situation Scale [5] was used, which measures the ease with which career urgency is provoked in a certain situation. It consists of 30 items. It has a four-factor structure, namely, “stagnation of career exploration,” “low evaluation from affiliation,” “upward comparison of the careers of friends and acquaintances,” and “lack of work–life balance.” Owing to the fact that the first factor contains 15 items, the number of items within the factors is skewed. Therefore, as with Ono [15], three items with a high load were extracted from each factor and used. The instruction content consisted of the following: “Below, the various situations regarding careers are presented. How frustrated do you think you would be in that situation? Please choose the one that you feel applies to you.” Participants were asked to answer on a four-point scale ranging from 1 (not at all) to 4 (very much).

## Results

### Comparison of scale scores

After calculating Cronbach’s reliability coefficient α for the subscales of each scale, the mean and standard deviation for each of the four groups (regular men, regular women, non-regular men, and non-regular women) were calculated according to gender and employment status, as well as a two-way ANOVA (Table 2). The results showed that the turnover intention (F(1, 396) = 5.38, p < 0.05, ηp2 = 0.01) had a significant interaction when the independent variables were combined. A simple main effect test revealed that the effect of gender was significant for regular employees (F(1, 396) = 8.76, p < 0.01, ηp2 = 0.02) and was significantly higher for women as opposed to men. Moreover, the effect of the employment status was significant for women (F(1, 396) = 4.34, p < 0.05, ηp2 = 0.01) and was significantly higher for regular employees as opposed to non-regular employees.

**Table 2:** Mean, SD, and analysis of variance results for each scale by group

	*α*	regular men (n = 111)		regular women (n = 111)		non-regular men (n = 71)		non-regular women (n = 107)		main effect		reciprocal action
		mean	*SD*	mean	*SD*	mean	*SD*	mean	*SD*	gender	employment status	gender × employment status
turnover intention		1.95	1.08	2.43	1.37	2.18	1.15	2.09	1.16	-	-	5.38^*^
feeling of being pressurized	0.92	2.45	1.00	2.77	1.01	2.80	1.01	2.60	0.99	-	-	6.39^*^
urge to develop one’s career	0.87	2.82	0.89	3.01	0.97	2.99	1.06	2.77	0.88	-	-	4.67^*^
concern for one’s career	0.84	3.22	1.00	3.60	0.83	3.32	0.99	3.49	0.86	3.06^**^	-	-
stagnation of career exploration	0.85	2.48	0.81	2.78	0.75	2.57	0.78	2.72	0.84	2.94^**^	-	-
low evaluation from affiliation	0.82	2.48	0.75	2.69	0.79	2.40	0.77	2.56	0.83	2.21^**^	-	-
upward comparison of the careers of friends and acquaintances	0.82	2.19	0.77	2.39	0.74	2.22	0.81	2.43	0.85	2.69^**^	-	-
lack of work–life balance	0.73	2.44	0.75	2.89	0.69	2.43	0.68	2.78	0.75	5.44^***^	-	-

***p < 0.001, **p < 0.01, *p < 0.05

In terms of career urgency, there was a significant interaction between the “feeling of being pressurized” (F(1, 396) = 6.39, p < 0.05, ηp2 = 0.02) and the “urge to develop one’s career” (F(1, 396) = 4.67, p < 0.05, ηp2 = 0.01). A simple main effect test revealed that the effect of gender on the “feeling of being pressurized” was significant for regular employees (F(1, 396) = 5.61, p < 0.05, ηp2 = 0.01) and was higher for women as opposed to men. Further, the effect of the employment status was significant for men (F(1, 396) = 5.16, p < 0.05, ηp2 = 0.01) and was significantly higher for non-regular employees than for regular employees. In terms of the “urge to develop one’s career,” the effect of the employment status tended to be significant for women (F(1, 396) = 3.72, p < 0.01, ηp2 = 0.01) and was higher for regular employees than for non-regular employees. No interaction was observed for “concern for one’s career,” and the results of the t-test showed a significant difference between genders (t(355.88) = 3.06, p < 0.10, r = 0.16), with higher numbers among women than among men.

With regard to the career urgency-provoking situations, no interactions were observed and the t-test revealed a significant difference between genders for all subscales, namely, “stagnation of career exploration” (t(398) = 2.94, p < 0.01, r = 0.15), “low evaluation from affiliation” (t(398) = 2.21, p < 0.05, r = 0.11), “upward comparison of the careers of friends and acquaintances” (t(398) = 2.69, p < 0.01, r = 0.13), and “lack of work–life balance” (t(398) = 5.44, p < 0.001, r = 0.26); men had higher values as when compared to women.

### Evaluation of model fit by covariance structure analysis

The paths were drawn from the four subscales of the career urgency-provoking situations to the three subscales of career urgency, and from the three subscales of career urgency to the turnover intention, based on the model that states that the tendency for impatience in situations such as “stagnation of career exploration,” “low evaluation from affiliation,” “upward comparison of the careers of friends and acquaintances,” and “lack of work–life balance” leads to turnover intention through career urgency, such as the “feeling of being pressurized,” “urge to develop one’s career,” and “concern for one’s career.” In addition, covariance was assumed between the four subscales of the Career Urgency-Provoking Situation Scale and the errors of the three subscales of career urgency. SPSS AMOS 26.0 was used for the covariance structure analysis.

**Fig. 1:**
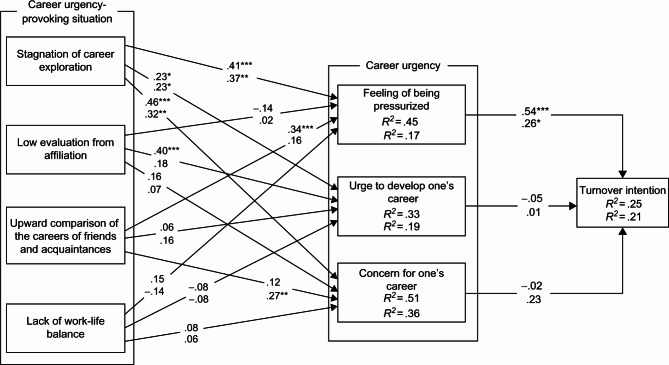
Simultaneous multi-population analysis (regular employees). ***p <.001, **p <.01, *p<.05. The upper row of path coefficients indicates males and the lower row indicates females. Descriptions of covariance and error variance were omitted

**Fig. 2:**
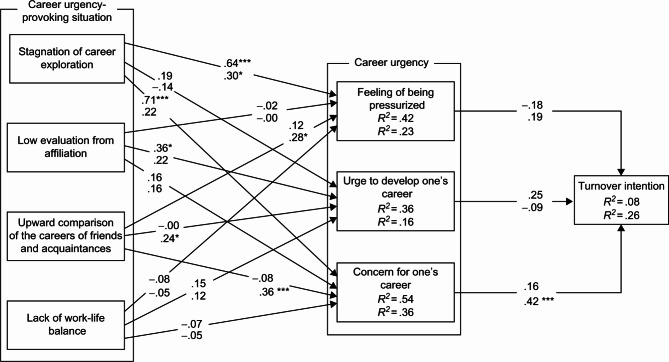
Simultaneous multi-population analysis (non-regular employees). ***p <.001, *p<.05. The upper row of path coefficients indicates males and the lower row indicates females. Descriptions of covariance and error variance were omitted.

First, the participants were divided into four groups—regular male employees, regular female employees, non-regular male employees, and non-regular female employees—and were then analyzed thereafter. After confirming the goodness of fit of the model in each group, a simultaneous multi-population analysis was performed. The results showed that goodness-of-fit index (GFI) = 0.992, adjusted goodness-of-fit index (AGFI) = 0.928, and root mean square error of approximation (RMSEA) = 0; thus, the fit of the model was good. Therefore, it is highly likely that the constructed path analysis model has a good fit for each group and that the placement invariance remains the same. Subsequently, the path coefficients were compared using equality constraints. Model 1 was set without equality constraints, Model 2 was set with equality constraints on all covariances, and Model 3 was set with the assumption that all covariances and path coefficients are equivalent in each group. Model 2 had an Akaike information criterion (AIC) value of 268.479, which is smaller than that of Model 1 (AIC = 268.915) and Model 3 (AIC = 271.971); this indicates that Model 2 fits the best. Figures 1 and 2 show standardized solutions as path coefficients for Model 1.

The results of the analysis revealed a significant path from “feeling of being pressurized” (β = 0.54, p < 0.001) to turnover intention in regular male employees. A significant path was revealed from “stagnation of career exploration” (β = 0.41, p < 0.001) and “upward comparison of the careers of friends and acquaintances” (β = 0.34, p < 0.001) to “feeling of being pressurized,” from “stagnation of career exploration” (β = 0.23, p < 0.05) and “low evaluation from affiliation” (β = 0.40, p < 0.001) to “urge to develop one’s career,” and from “stagnation of career exploration” (β = 0.46, p < 0.001) to “concern for one’s career.” The results of the analysis revealed a significant path from the “feeling of being pressurized” (β = 0.26, p < 0.05) to turnover intention in regular female employees. A significant path was revealed from “stagnation of career exploration” (β = 0.37, p < 0.01) to “feeling of being pressurized,” from “stagnation of career exploration” (β = 0.23, p < 0.05) to “urge to develop one’s career,” and from “stagnation of career exploration” (β = 0.32, p < 0.01) and “upward comparison of the careers of friends and acquaintances” (β = 0.27, p < 0.01) to “concern for one’s career.”

Among the non-regular male employees, no significant effect on turnover intention was observed in any of the subscales of career urgency. However, a significant path was revealed from “stagnation of career exploration” (β = 0.64, p < 0.001) to “feeling of being pressurized,” from “low evaluation from affiliation” (β = 0.36, p < 0.05) to “urge to develop one’s career,” and from “stagnation of career exploration” (β = 0.71, p < 0.001) to “concern for one’s career.” The results of the analysis revealed a significant path from “concern for one’s career” (β = 0.42, p < 0.001) to turnover intention in non-regular female employees. Moreover, a significant path was revealed from “stagnation of career exploration” (β = 0.30, p < 0.05) and “upward comparison of the careers of friends and acquaintances” (β = 0.28, p < 0.05) to “feeling of being pressurized,” from “upward comparison of the careers of friends and acquaintances” (β = 0.24, p < 0.05) to “urge to develop one’s career,” and from “upward comparison of the careers of friends and acquaintances” (β = 0.36, p < 0.001) to “feeling of being pressurized.”

Further, when the test statistic for the difference between the path coefficients was calculated, a value of 3.26 was revealed for the path from “stagnation of career exploration” to “concern for one’s career,” showing a significant difference between regular female employees and non-regular male employees.

## Discussion

### Characteristics of scale scores by gender and employment status

The turnover intention was significantly higher for women than for men among regular employees, supporting the results of Nakamura [34] as well as Stroh et al. [35], which state that women’s turnover intention is higher than that of men. However, some studies show higher values for men [36], and in others, differences in turnover intention based on gender were not observed (e.g., Ono and Yukawa [33]; Vigoda [37]). Although studies that show no differences between genders are more common, no consensus has been reached in this regard. Further, among women, the values were significantly higher for regular employees than for non-regular employees. This is thought to be because new graduates who are unable to obtain a good job initially will also find it difficult to subsequently change to a job with better working conditions [9]; women who were temporary clerical workers in their first job tend to become involuntary non-regular employees [13].

With regard to career urgency, the “feeling of being pressurized” was significantly higher among women than among men in relation to regular employees, and was significantly higher among non-regular employees as opposed to regular employees for men. The “feeling of being pressurized” is a negative aspect of career urgency, and the results showed that even among regular employees, women tended to exhibit negative feelings more than men. The determining factors for women’s career development are grouped into three categories, namely, “women’s own factors,” “family factors,” and “workplace factors”; furthermore, research has shown that there are more intervening factors for women than for men [32]. This shows that women are more likely to face conflicts in their career development, which may result in their feeling hopeless. Moreover, a likelihood exists that the awareness of the division of labor by gender roles is contributing to non-regular male employees having more negative feelings of career urgency than regular male employees. In Japan, more than 30% of people still agree with the division of labor by gender roles [38]. However, in terms of the occupations of young adult male workers who have graduated from university or graduate school, clerical jobs are decreasing and blue-collar jobs are increasing [12]. If a new graduate cannot find a job with good working conditions, it is difficult to change to a job with better conditions [9]. Therefore, men who have graduated from university or graduate school but become non-regular employees are likely to feel hopeless with regard to their careers. The positive aspect of career urgency, the “urge to develop one’s career,” was significantly higher for female regular employees than for female non-regular employees. Therefore, women who are regular employees are more driven to quickly develop their careers to achieve their goals. Since women who were temporary clerical workers in their first job tend to become involuntary non-regular employees [13], women who are non-regular employees may find it difficult to develop their careers and are less likely to develop positive urges. In addition, no interaction was observed in the “concern for one’s career,” and the values were significantly higher for women than for men. In other words, regardless of the employment status, women are more concerned about their current and future careers. This is a result of women’s career development being influenced by more factors than that of men [32]. Predicting their future career is difficult for women; thus, women are more prone to having more anxiety and concern about their future, and about whether they should continue with the job they currently have.

In terms of the career urgency-provoking situations, there was no interaction between “stagnation of career exploration,” “low evaluation from affiliation,” “upward comparison of the careers of friends and acquaintances,” and “lack of work–life balance”; the scores were significantly higher for women than for men. In the study by Ono and Yukawa [33], which targeted regular employees in their 20‒40 s with regard to their age, only the “upward comparison of the careers of friends and acquaintances” did not have a gender difference, but in the other three factors, the values for women were higher than those for men. This is thought to be because even if one has the ability and motivation, developing a career is difficult if the factors at work and home are not ideal [33]. This study also revealed that women were more likely to experience career urgency in various situations as opposed to men.

### Examination of model

In this study, the model that states that the tendency for impatience in situations such as “stagnation of career exploration,” “low evaluation from affiliation,” “upward comparison of the careers of friends and acquaintances,” and “lack of work–life balance” leads to turnover intention through career urgency, such as the “feeling of being pressurized,” “urge to develop one’s career,” and “concern for one’s career” was analyzed by grouping the participants in accordance with the gender and employment status. The goodness of fit of the model was found to be good, and the following points were clarified:

First, among both male and female regular employees, “stagnation of career exploration” was found to promote the “feeling of being pressurized,” “urge to develop one’s career,” and “concern for one’s career”; moreover, the “feeling of being pressurized” was found to promote turnover intention. In Ono’s [15] study, regular employees in their 20s were analyzed. The results showed that the “stagnation of career exploration” promotes the “feeling of being pressurized” and “concern for one’s career,” and that the “feeling of being pressurized” promotes turnover intention. These results are generally consistent with those of the present study. However, the path shown by Ono [15] from “low evaluation from affiliation” to “urge to develop one’s career” and “upward comparison of the careers of friends and acquaintances” to “feeling of being pressurized” was significant only for regular male employees in the present study. The path from “upward comparison of the careers of friends and acquaintances” to “concern for one’s career,” which was not shown in Ono [15], was significant only for women in the present study. This study shows that there are some gender differences in the relationship between career urgency-provoking situations and career urgency, even among regular employees. Yamauchi [39] constructed a measurement scale for achievement-related motivation within the framework of Atkinson [40], who viewed achievement tendencies as the combined forces of achievement motivation and failure avoidance, and reported that men had a stronger desire for success than women did. It has also been reported that the desire for social achievement is higher in men than in women [5]. The competitive achievement motive, which corresponds to the desire for social achievement, is an achievement motive that aims to be valued by society by winning over others [41]. Therefore, it is thought that when men receive a low evaluation from their affiliation, this leads to the urge to quickly build a career that is recognized by those around them.

Second, among non-regular employees, the only aspect that men and women had in common was the path from “stagnation of career exploration” to “feeling of being pressurized.” Since this was common to both the regular male and female employees, the fact that impatience was felt when one’s career exploration stagnates provokes the “feeling of being pressurized” and is likely a characteristic of young adult workers as a whole. Further, the results showed that the “stagnation of career exploration” prompted “concern for one’s career” and “low evaluation from affiliation,” which prompted the “urge to develop one’s career” only in men, and was also the case with regular male employees. As mentioned above, this is thought to be a result of the difference in the strength of the desire for success between men and women. However, the results showed that “upward comparison of the careers of friends and acquaintances” promoted the “feeling of being pressurized,” “urge to develop one’s career,” and “concern for one’s career” only in women. Although the path to “urge to develop one’s career” was also present for regular female employees, the results showed that the impatience felt by non-regular female employees when they compare their own careers with those of their friends and acquaintances who are in a more desirable state than their own tends to provoke the career urgency. Moreover, “concern for one’s career” led to turnover intention only among non-regular female employees. The “concern for one’s career” is a vague feeling that can be both negative and positive [19], but it is not strong. For non-regular female employees, quitting a job may not require a great amount of determination, and they are likely to develop the turnover intention as a means of resolving doubts about whether they can continue their careers in the same way in their current situation. Among non-regular male employees, no path was found to lead to turnover intention from career urgency. The two studies of Ono, which targeted regular employees in their 20s [15] and in their 20‒40 s [23] showed that the “feeling of being pressurized” promotes turnover intention. In the present study, the same results were obtained for regular male and female employees, suggesting that this relationship is a characteristic of regular employees and does not apply to non-regular employees.

The percentage of those for whom retirement or expiration of the employment contract is the reason for leaving a job was higher for non-regular employees than for regular employees among both men and women [42]. The factors influencing the turnover intention may differ from those of regular employees because the latter can work until retirement and decide when to leave their jobs independently, whereas non-regular employees are restricted by the length of their contracts.

## Conclusions

This study’s results show that young adult workers’ feelings of career urgency and turnover intention differ by gender and employment type; women are more likely to develop career urgency in various situations than men. The model states that the tendency for impatience in situations such as “stagnation of career exploration,” “low evaluation from affiliation,” “upward comparison of the careers of friends and acquaintances,” and “lack of work–life balance” leads to turnover intention through career urgency, such as the “feeling of being pressurized,” “urge to develop one’s career,” and “concern for one’s career.” This model was analyzed by grouping the participants in accordance with gender and the employment status. The results of both male and female regular employees were consistent with Ono’s [15] finding that the feeling of impatience when one’s career exploration stagnates leads to turnover intention through the “feeling of being pressurized.” However, for both male and female non-regular employees, the tendency for impatience is a result of the stagnation of career exploration, which promotes the “feeling of being pressurized.” This does not lead to turnover intention. Furthermore, the results showed that in the case of non-regular male employees, the career urgency does not lead to turnover intention, and in the case of female non-regular employees, the tendency for impatience when comparing their own career with those of friends and acquaintances who are in a more desirable state than their own leads to turnover intention through “concern for one’s career.”

Since around 2000, workers in Japan have been required to develop their careers autonomously, but there are still some workers whose career exploration and development are not going smoothly. This study’s results suggest that stagnation in one’s career exploration may arouse career urgency, which may, in turn, lead to turnover. Retention management has become an issue for many companies, and it may be necessary to have a system that not only requires regular employees to have autonomous careers but also supports their career development. It is also necessary to look at the careers of non-regular employees. Since this study showed differences in the psychology of non-regular men and women, it is important to understand the characteristics of each group, rather than lumping all non-regular employees together.

### Limitations

This study had a few limitations. First, the survey participants in this study were registered monitors of an Internet research firm. With regard to Internet surveys using registered monitors, issues of sample representativeness and measurement problems have been pointed out [43]. In the future, efforts should be made to obtain as many biases and characteristics as possible as a sample from the research firm and to tighten the criteria for excluding respondents with unserious attitudes. In addition, this study examined the relationship between career urgency and turnover intention in terms of gender and employment status. In reality, however, other variables may also exist. Future studies should consider not only gender and employment status, but also marital status and the presence or absence of children. In addition, in terms of non-regular employees, a detailed study is required that accounts for variables such as whether they are non-regular employees of the involuntary type and wish to change jobs to work as regular employees.

## Data Availability

The data sets generated or analyzed in this study are not publicly available but are available from the corresponding author upon reasonable request.

## Abbreviations

GHQ: General Health Questionnaire
PHQ: Patient Health Questionnaire

## References

[1] Ministry of Labor. Analysis of labor economy 1998 edition; 1998.

[2] Hall DT (1996). Protean careers of the 21st century. AMP.

[3] Arthur MB, Rousseau DM. The boundaryless career: a new employment principle for a new organizational era. Oxford University Press; 1996.

[4] Kossek EE, Roberts K, Fisher S, Demarr B (1998). Career self-management: a quasi-experimental assessment of the effects of a training intervention. Pers Psychol.

[5] Ono H, Yukawa S (2008). Career-urgency and turnover intention of white-collar workers in twenties. Jpn J Couns Sci.

[6] Ono H, Okada M (2014). Structure of career-urgency, the action which arises by it, and alleviation process of career-urgency: explorative study based on interviews with white-collar workers in twenties. Career Des.

[7] Ministry of Health, Labor and Welfare/Ministry of Education, Culture, Sports, Science and Technology. Survey on employment status of university graduates in 2021 (as of April 1, 2022). 2022. https://www.mhlw.go.jp/content/11805001/000939599.pdf. Accessed 28 Sep 2022.

[8] Ministry of Health, Labor and Welfare. Overview of the 2018 Youth Employment Survey. 2019. https://www.mhlw.go.jp/toukei/list/dl/4-21c-jyakunenkoyou-h30_gaikyou.pdf. Accessed 26 Sep 2022.

[9] Mizuochi M (2020). Impact of employment status at the time of graduation on subsequent employment and income. J Personal Fin Econ.

[10] Sakai T, Higuchi Y (2005). Freelance workers: employment, income, marriage, Childbirth. Jpn Lab Stud.

[11] Ministry of Education, Culture, Sports, Science and Technology. 2021 school basic survey (final version). 2021. https://www.mext.go.jp/content/20211222-mxt_chousa01-000019664-1.pdf. Accessed 27 Sep 2022.

[12] Kosugi R (2017). Transformation of jobs among university graduates. Jpn J Higher Educ Res.

[13] Kurokawa S (2020). Promotion of women’s participation and involuntary non-regular employment. Annual Rep Sociol Tokyo Woman’s Christ Univ Ann Sociol.

[14] Ministry of Health, Labor and Welfare. Status of turnover of new graduates. 2021. https://www.mhlw.go.jp/content/11652000/000845829.pdf. Accessed 28 Sep 2022.

[15] Ono H (2016). Career-urgency and career-urgency-provoking situation among young adult workers: how these two concepts are relevant with turnover intention. Jpn J Couns Sci.

[16] Friedman M, Rosenman RH (1959). Association of specific overt behavior pattern with blood and cardiovascular findings: blood cholesterol level, blood clotting time, incidence of arcus senilis, and clinical coronary artery Disease. JAMA.

[17] Chapman AH, Chapman MC. Harry Stack Sullivan’s concepts of personality development and psychiatric Illness. Brunner/Mazel; 1980.

[18] Lazarus RS. Stress and emotion: a new synthesis. Springer Publishing Company; 1999.

[19] Ono H, Okada M (2014). Structure of career-urgency with young adult workers: development of a Career-urgency scale. Jpn Assoc Ind Organ Psychol Res J.

[20] Yamamoto S (1992). An examination of two distinct aspects of anxiety in adolescence. Shinrigaku Kenkyu.

[21] Nakagawa Y, Daibo I. Guide to the Japanese version of the GHQ Mental Health Questionnaire. Nihon Bunka Kagakusha; 1985.

[22] Ono H (2022). Relationship between career self-reliance, turnover intention, and career urgency of workers. Jpn Assoc Ind Organ Psychol Res J.

[23] Ono H (2018). Career-urgency and turnover intention of workers: comparison between high and low work engagement groups. Jpn J Couns Sci.

[24] Ono H (2015). Behavior caused by career-urgency of young adult workers. Career Des.

[25] Ono H (2019). Career urgency of young adult workers and leader-member/team-member exchange relationships – focusing on the relationship with turnover activities. Career Des.

[26] Ono H (2017). Factors alleviating career-urgency of young adult workers. Jpn J Couns Sci.

[27] Yamamoto I. Hopes and reality of non-regular workers: reality of involuntary non-regular employment. RIETI Discussion Paper Series 11. 2011. https://www.rieti.go.jp/jp/publications/dp/11j052.pdf, J-052. Accessed 26 Sep 2022.

[28] Matsuyama K (2010). Job attitudes and mental health of nonpermanent employees. Jpn J Admin Sci.

[29] Allen NJ, Meyer JP (1990). The measurement and antecedents of affective, continuance and normative commitment to the organization. J Occup Psychol.

[30] Gakovic A, Tetrick LE (2003). Perceived organizational support and work status: a comparison of the employment relationships of part-time and full-time employees attending university classes. J Organiz Behav.

[31] Cabinet Office. White paper on gender equality. 2013. 2013. http://www.gender.go.jp/about_danjo/whitepaper/h25/zentai/. Accessed 1 Apr 2022.

[32] Wakabayashi M (1985). Structure of career development in women – its possibilities and limits. Lab Stud.

[33] Ono H, Yukawa S (2010). Career-urgency and turnover intention of white-collar workers: comparison by age. Jpn J Couns Sci.

[34] Nakamura J. Who leaves companies? Job turnover tendency and matching within a company. In: Economics of “Turnover” – Choosing the Right Job and Human Resource Development. Inoki, T, editor. Research Institute for Advancement of Living Standards, editor. Toyo Keizai Inc; 2001. p. 21–44.

[35] Stroh LK, Brett JM, Reilly AH (1996). Family structure, glass ceiling, and traditional explanations for the differential rate of turnover of female and male managers. J Vocat Behav.

[36] Turnley WH, Feldman DC (2000). Re-examining the effects of psychological contract violations: unmet expectations and job dissatisfaction as mediators. J Organiz Behav.

[37] Vigoda E (2000). Organizational politics, job attitudes, and work outcomes: exploration and implications for the public sector. J Vocat Behav.

[38] Cabinet Office. Public opinion survey on gender equal society. 2019. https://survey.gov-online.go.jp/r01/r01-danjo/index.html. Accessed 1 Nov 2022.

[39] Yamauchi H (1980). Analysis of measurement scales for achievement-related motivation. Jpn J Educ Psychol.

[40] Atkinson JW (1957). Motivational determinants of risk-taking behavior. Psychol Rev.

[41] Horino M, Mori K (1991). Factors of achievement motivation mediating the relationship between depression and social support. Japanese J Educ Psychol.

[42] Ministry of Health, Labor and Welfare. Analysis of the Labor Economy, 2022 Edition – Issues in Promoting Labor Mobility through Supporting Workers’ Independent Career Development. https://www.mhlw.go.jp/stf/wp/hakusyo/roudou/21/21-1.html. Accessed 28 Sep 2022.

[43] Science Council of Japan. Toward Effective Academic Use of Web Surveys. Committee on Sociology (Subcommittee to Study Issues of Web Surveys). https://www.scj.go.jp/ja/info/kohyo/pdf/kohyo-24-t292-3. Pdf. 2020. Accessed 24 Oct 2023.

